# Investigating the Associations Between Hmga2 Overexpression, R-Loop Reduction, and Bone Loss in Aging Mice

**DOI:** 10.3390/medicina61050820

**Published:** 2025-04-29

**Authors:** Yangyang Cao, Yantong Wang, Dengsheng Xia

**Affiliations:** 1 Laboratory of Molecular Signaling and Stem Cells Therapy, Beijing Key Laboratory of Tooth Regeneration and Function Reconstruction, School of Stomatology, Capital Medical University, No. 4 Tiantanxili, Dongcheng District, Beijing 100050, China; yangyangcao@mail.ccmu.edu.cn (Y.C.); wyt@mail.ccmu.edu.cn (Y.W.); 2 Department of General Dentistry and Integrated Emergency Dental Care, Beijing Stomatological Hospital, Capital Medical University, Beijing 100050, China

**Keywords:** aging, Hmga2, R-loop, bone marrow-derived mesenchymal stem cells (BMSCs), osteogenic differentiation

## Abstract

*Background and Objectives:* Aging-related bone loss still lacks interventions. As bone marrow-derived mesenchymal stem cells (BMSCs) undergo aging, R-loop-induced DNA replication stress impairs the osteogenic ability of BMSCs. High-mobility group A-2 (Hmga2) acts as a DNA-binding protein, and the understanding of its underlying mechanisms is crucial for developing effective preventive and therapeutic strategies. *Materials and Methods:* Aging mice were used as the experimental model, and mouse BMSCs were isolated from their femurs. Hmga2 was achieved through specific gene delivery methods. R-loop formation was detected using dot blotting, chromatin immunoprecipitation (ChIP), and DNA–RNA immunoprecipitation (DRIP) assays. Osteogenic differentiation was evaluated. *Results:* R-loops were highly accumulated in aging BMSCs. Notably, the key regulator Hmga2 reversed the accumulation of R-loops in aging BMSCs. Hmga2 overexpression significantly decreased the senescence and improved the osteogenic differentiation of aging mBMSCs. Mechanistically, R-loop-forming sequence (RLFS) regions were confirmed in key osteogenesis-related genes, including runt-related transcription factor 2 (Runx2). Hmga2 bound to the RLFS region of Runx2 and promoted its expression by reducing the R-loop level. More, Hmga2 treatment delivered via the AAV system effectively decreased bone loss in aging mice and increased the serum bone turnover biomarkers and collagen remodeling. *Conclusions:* Our study demonstrates that Hmga2 acts as an activator of aging BMSCs, significantly promoting their osteogenic ability by eliminating the aging-induced DNA replication stress caused by R-loops. Our findings provide new insights into the mechanisms of aging-related bone loss, suggesting that Hmga2 may be a new strategy for alleviating the bone loss phenotype in aging individuals.

## 1. Introduction

Bone loss is the most common type of aging-related bone defect, which has a long-term influence on quality of life, high health costs, and social burdens [1,2]. Recent pertinent data demonstrate that aging-related bone loss, such as osteopenia and osteoporosis, increased in prevalence from 21.47% to 56.23% and from 0.89% to 17.23%, respectively [3]. Aging-related bone loss results in insufficient jawbone mass in the elderly, which affects the implementation and quality of implant surgery. A useful solution for improving bone homeostasis in aging bodies is still lacking. Bone marrow-derived mesenchymal stem cells (BMSCs) constitute a key cell population in bone marrow and are associated with the maintenance of bone homeostasis [4]. One study reported that the osteogenic ability of aging BMSCs is impaired due to the accumulation of aging-related stressors, such as telomere attrition and DNA replication stress [5]. During the aging process, exposure to these stressors leads to an aging-dependent decline in the multipotent properties of BMSCs, including cell viability, senescence-associated marker levels, and osteogenic differentiation ability [6]. The modulation of multipotent properties in aging BMSCs might provide a solution to slow or reverse aging. Therefore, it is still necessary to clarify aging-associated DNA replication stress and the underlying key regulatory factors in aging BMSCs.

The transcribed single-stranded RNA inevitably invades the template DNA strands and rehybridizes with double-stranded DNA to form a DNA/RNA structure, which is referred to as the R-loop [7]. A high level of RNA transcription makes MSCs particularly vulnerable to perturbations in gene expression, which is associated with increased R-loop formation [8]. Although reports on R-loops and aging BMSCs are very limited, genomic R-loop coverage significantly increases during the aging process, and the age-related distribution is associated with specific features, such as transcript levels and GC content [8]. R-loop formation is closely related to DNA replication stress and blocks the progression of the replication fork in key transcription factors [7]. Runt-related transcription factor 2 (Runx2) is known as the key transcription factor for osteogenic differentiation. However, the details of the R-loops in these key osteogenesis-related genes are still lacking. How R-loops influence the osteogenic differentiation of BMSCs is still unclear.

R-loops are closely associated with DNA-binding proteins. A previous study revealed the different families of DNA-binding proteins that regulate R-loops, including HMGA family proteins [9]. High-mobility group A-2 (Hmga2) is a non-histone chromosomal HMGA family protein that alters chromatin structure through DNA binding [10]. Hmga2 contains three separate DNA-binding domains that consist of 8 or 9 amino acids and have high affinity for short AT-rich sequences [11]. Hmga2 reduces the SA-β-gal level and improves the migration ability of aging-derived BMSCs (24-month-old rats) [12]. Hmga2 mutation strongly reduces the bone phenotype and body size of mice [11,13]. Hmga2 knockout suppresses the differentiation of preosteoblast MC3T3-E1 cells into osteoblasts [14]. Hmga2 overexpression rescues the suppressed osteoblast differentiation of mBMSCs and increases new bone regeneration in injured mouse femurs [15,16,17]. These results indicate that Hmga2 is related to the nuclear abnormalities of aging MSCs and might play a pivotal role in bone regeneration. However, how Hmga2 regulates the transcription of key osteogenic genes in aging BMSCs via R-loops remains unclear.

In this study, the role and mechanism of Hmga2 in the osteogenic differentiation of aging mBMSCs were investigated. Hmga2 significantly improved the osteogenic differentiation ability of aging mBMSCs by eliminating the highly accumulated R-loop formation of key osteogenesis-related genes, including runt-related transcription factor 2 (Runx2), osterix (Osx), alkaline phosphatase (Alpl), collagen type I alpha 1 (Col1a1), osteocalcin (Ocn), and bone morphogenetic protein 2 (Bmp2).

## 2. Materials and Methods

### 2.1. Animals

C57BL/6J mice (6–8 weeks old) were purchased from Cyagen Biosciences (Suzhou, China). The mice were carefully cared for and raised until they reached 20 months of age. The animal care and experimental procedures were performed following the guidelines of the Beijing Experimental Animal Management Ordinance (Approval of Animal Ethical and Welfare: KQYY-2022). Male mice (20 months old) exhibiting normal grooming and walking behavior were obtained and selected for this study.

### 2.2. Cell Isolation and Culture

Aging mBMSCs were obtained from the bone marrow of the femurs of aging mice. Briefly, single-cell suspensions were obtained by repeated blowing, and monocytes were collected with Percoll (specific gravity of 1.082; Invitrogen, Carlsbad, CA, USA) and then cultured in a humidified environment containing 5% CO_2_ at 37 °C. The culture medium was changed every 3 days and consisted of alpha-modified Eagle’s medium (α-MEM) (Invitrogen) supplemented with 15% fetal bovine serum (FBS; Invitrogen), 100 U/mL penicillin, and 100 μg/mL streptomycin (Invitrogen). Passage 3–5 cells were used for analysis.

To package viral constructs, human embryonic kidney 293T cells were maintained in Dulbecco’s modified Eagle’s medium (DMEM) (Invitrogen) supplemented with 10% FBS, 100 U/mL penicillin, and 100 μg/mL streptomycin (Invitrogen).

### 2.3. Plasmid Construction and Viral Infection

As previously described [18], plasmids containing short hairpin RNA (shRNA) with a specific target sequence of Hmga2 (pLKO.1 lentiviral vector; Addgene, Cambridge, MA, USA) and a full-length gene sequence with an HA tag of Hmga2 (HA-pQCXIN retroviral vector; BD Biosciences, Franklin Lakes, NJ, USA) were constructed. Then, the virus was packaged in 293T cells, and the aging mBMSCs were infected with the retro- or lentivirus. The control shRNA (Consh, Houston, TX, USA) was purchased from Addgene (Cambridge, MA, USA). The target shRNA sequence of Hmga2 (Hmga2sh) was as follows: 5′-AGACCCAGAGGAAGACCCAAA-3′.

### 2.4. Real-Time Reverse Transcriptase–Polymerase Chain Reaction (Real-Time RT–PCR)

Briefly, total RNA was extracted from aging mBMSCs, and cDNA synthesis was performed as previously described [18]. Real-time RT–PCR was performed via the standard protocol of the CFX384 Touch Real-Time PCR Detection System (Bio-Rad, Hercules, CA, USA), QuantiTect SYBR Green PCR Kit (Qiagen, Hilden, Germany), and an Icycler iQ Multicolor Real-Time RT–PCR detection system. The sequences of the primers used in this study are listed in Supplementary Table S1.

### 2.5. Western Blot

The total protein of aged BMSCs was extracted as previously described [18]. The nuclear protein was extracted according to the manufacturer’s recommendations (NE-PER™ Nuclear and Cytoplasmic Extraction Kit; Catalog No. 78833; Thermo Scientific, Waltham, MA, USA). The extracted protein was analyzed using SDS–polyacrylamide gel electrophoresis [18]. Briefly, the primary antibodies used in this study were anti-HA (catalog no. ab9110; Abcam, Cambridge, UK), anti-Hmga2 (catalog no. ab207301; Abcam), anti-Runx2 (catalog no. ab192256; Abcam), anti-collagen-1 (catalog no. ab316222; Abcam), and anti-BSP (catalog no. EPR23243-30; Abcam). Anti-GAPDH (catalog no. ab8245; Abcam) and anti-histone H3 (catalog no. ab1791; Abcam) were used as total and nuclear protein markers, respectively.

### 2.6. Dot Blotting

The genomic DNA of aging mBMSCs was extracted according to the manufacturer’s protocol for the GenElute™ Mammalian Genomic DNA Miniprep Kit (catalog No. G1N70, Millipore, Darmstadt, Germany). Approximately 2 μg of each DNA sample was incubated with 20× SSC and 37% formaldehyde at 65 °C for 15 min and then immediately transferred to ice. The DNA sample mixture was placed on the activated PVDF membrane, subjected to UV crosslinking, and irradiated for 15 min. After being washed with 1× TBST solution, the PVDF membrane was incubated in 5% non-fat milk for 2 h and then incubated with the primary antibody anti-S9.6 (catalog no. MABE1095; Millipore) at 4 °C overnight. The film was exposed to HRP-conjugated chemiluminescence, and the results were recorded.

### 2.7. Senescence-Associated β-Galactosidase (SA-β-Gal) Staining

The Senescence β-Galactosidase Staining Kit protocol (catalog No. 9860, Cell Signaling Technology, Danvers, MA, USA) was used and SA-β-gal in aging mBMSCs was detected. Briefly, aging mBMSCs were grown on coverslips and fixed with 4% paraformaldehyde. After washing, β-Gal staining solution was added, and the mixture was incubated at 37 °C for 2 h. Microscopy (Olympus, Tokyo, Japan) was used to examine the degree of staining, and Image-Pro Plus 6.0 (ImageJ-FIJI-Trmk, Tokyo, Japan) was used to measure the percentage of positively stained cells.

### 2.8. Telomerase Reverse Transcriptase (TERT) Level Analysis

The TERT level was assessed via a TERT enzyme-linked immunosorbent assay (ELISA, Helsinki, Finland) kit according to the manufacturer’s recommendations (Teloeras Reverse ELISA Kit, CUSABIO, Houston, TX, USA). In brief, a 200 μg/100 μL protein sample was added to the analysis well and incubated at 37 °C for 2 h. Then, 100 μL of biotin-conjugated antibody, 100 μL of HRP-conjugated streptavidin, and 90 μL of TMB substrate were added. Finally, 50 μL of Stop Solution was added to each well, and the optical density of each well was read at 450 nm within 5 min.

### 2.9. Alkaline Phosphatase Activity and Alizarin Red Detection

For osteogenic induction, 2 × 10^5^ cells were plated in each well of a 6-well plate, and a StemPro osteogenesis differentiation kit (Invitrogen) was used. For the ALP activity assay, the cells were induced for 5 days and then incubated with 1× BCIP/NBT buffer (Sigma, Kanagawa, Japan). For alizarin red staining, the cells were induced for 2 weeks and then stained with 2% alizarin red (Sigma). For Ca^2+^ concentration analysis, 10% cetylpyridinium chloride (CPC) was used to destain the alizarin red for 30 min at room temperature. The absorbance at 562 nm was measured, and the concentration was determined.

### 2.10. Bioinformatic Analysis

The full-length DNA and mRNA sequences of Runx2 were obtained from the NCBI website (https://www.ncbi.nlm.nih.gov; accessed on 26 March 2023). The R-loop-forming sequence (RLFS) of the Runx2 gene was predicted via the R-Loop DB website (http://R-Loop.org/; accessed on 26 March 2023) and the QmRLFS-Finder website (http://R-Loop.org/?pg=qmrlfs-finder; accessed on 26 March 2023). The bioinformatic analysis was performed via the UCSC website (https://genome.ucsc.edu; accessed on 26 March 2023).

### 2.11. Chromatin Immunoprecipitation (ChIP) and DNA–RNA Immunoprecipitation (DRIP) Assays

A ChIP assay kit (catalog no. MABE1095; Millipore) was used according to the manufacturer’s protocol. Briefly, each reaction employed 2 × 10^6^ aging mBMSCs, which were cross-linked with formaldehyde. After being incubated at 4 °C and centrifuged, the cells were resuspended in lysis buffer (1% Triton X-100; 50 mM KOH, pH 7.5; 1 mM EDTA, pH 8; and 140 mM NaCl; protease inhibitor) and lysed via ultrasonication. The extracted cells were subsequently centrifuged and resuspended in lysis buffer supplemented with 0.3% SDS. IP extraction was subsequently performed.

For DRIP, anti-S9.6 (catalog no. MABE1095; Millipore) was used to detect the RNA/DNA hybrids and to measure R-loop levels. IP extraction (1 mg/mL) was precleared, and the mixture was incubated with protein A beads (Invitrogen) overnight at 4 °C. For ChIP, anti-Hmga2 (catalog no. ab207301; Abcam) was used. Normal rabbit IgG (catalog no. ab172730; Abcam) and normal mouse IgG (catalog no. sc-2025; Santa Cruz Biotechnology, Dallas, TX, USA) were used as negative controls. In addition, 5% IP extraction was used as the input. The beads were washed for 10 min at 4 °C, followed by lysis buffer, high NaCl buffer (lysis buffer with 500 mM NaCl), LiCl buffer (250 mM LiCl; 1 mM EDTA; 10 mM Tris-HCl, pH 8.0; 1% sodium deoxycholate and NP-40), and TE buffer (pH 8.0). Bead-bound DNA was eluted twice using 150 μL of elution buffer (1% SDS; 50 mM Tris-HCl, pH 7.5; and 10 mM EDTA, pH 8.0) for 10 min and then treated with proteinase K (0.75 mg/mL; Invitrogen) overnight at 65 °C. Finally, DNA was extracted via the phenol–chloroform method. The precipitated DNA was quantified via real-time RT–PCR analysis. The final data are expressed as the percentage of input DNA.

### 2.12. Adeno-Associated Virus (AAV)-Mediated Gene Transduction in Aging Mice

Briefly, the DNA sequence of mouse Hmga2 was obtained and fused to the N-terminus of the rAAV9 vector, which was used for rAAV production (6 × 10^12^ GC/mL). Twenty aging mice (male, 20 months old, Cyagen Biosciences, C57BL/6J) were randomly divided into two groups: the rAAV9-Vector injection group and the rAAV9-HA-Hmga2 injection group. Anesthesia with 90 mg/kg ketamine and xylazine 10 mg/kg 1% sodium pentobarbital (90 mg/kg) was conducted via an intraperitoneal injection, and approximately 100 μL of rAAV9 virus (1 × 10^11^ GC per site) was injected into the proximal joint end of the femur of the aging mice. After injection for 3 weeks, the fluorescence expression in individual tissues of mice immobilized by inhalation anesthesia of isoflurane was monitored via IVIS-100 optical imaging. After injection for 12 weeks, the fluorescence expression in individual tissues of mice immobilized under inhalation anesthesia with isoflurane was monitored via IVIS-100 optical imaging.

### 2.13. Microcomputed Tomography (Micro-CT) Analysis

For trabecular and cortical bone mass and microarchitecture evaluation, mouse femur bones were harvested, fixed overnight in 4% paraformaldehyde, and then scanned for micro-CT analysis (eXplore Locus SP; GE Healthcare, Chicago, IL, USA). The scanning parameters were 70 kV and 80 μA, and the thickness of the tomography was 6 μm. A three-dimensional structure of each femur bone was established. Briefly, the data of the trabecular bone area of interest (AOI) were obtained from a 3 mm region of the distal metaphysis, approximately 0.3 mm away from the epiphysis. The data of the cortical AOI were obtained from the midshaft, approximately 4 mm away from the epiphysis. The data were analyzed with the DataView software (version 2.1.2), and the quantification parameters of bone mineral density (BMD) and bone volume per tissue volume (BV/TV) were determined using the CTan software (version 1.15).

### 2.14. Serum Enzyme-Linked Immunosorbent (ELISA)

Approximately 500 μL of whole peripheral blood was collected from the retro-orbital venous plexus of each mouse before necropsy. The serum was isolated through centrifugation at 1000× *g* for 10 min followed by 5000× *g* for 10 min to remove cell debris at 4 °C. Bone turnover-sensitive biomarkers, including the bone formation marker procollagen I N-terminal peptide (PINP) and the bone resorption marker cross-linked C-telopeptide of type 1 collagen (CTX-1), were detected in serum using murine ELISA kits according to the manufacturers’ instructions (R&D Systems, Minneapolis, MN, USA).

### 2.15. Histology and Histomorphometry

The tissue slices were deparaffinized and hydrated with distilled water. For hematoxylin–eosin (HE) staining, deparaffinized tissue slices were incubated with Weigert’s iron hematoxylin solution at room temperature (RT) for 5 min, acidified within seconds, and incubated in 0.1% eosin staining solution for 2 min. For picrosirius red staining, the Picrosirius Red Stain Kit (MaoKang Bio, Shanghai, China) was used. Briefly, deparaffinized tissue slices were adequately incubated with picrosirius red solution at RT for 60 min. The nucleus was dyed with Mayer’s hematoxylin solution for 10 min. For Masson’s trichrome staining, a Masson’s Staining Kit (Sigma–Aldrich, St. Louis, MO, USA) was used. Briefly, deparaffinized tissue slices were incubated with Weigert’s iron hematoxylin solution at RT for 5 min and acidified within seconds. Then, the slices were incubated with Ponceau S at RT for 5 min and rinsed quickly with 0.5% glacial acetic acid. The sections were washed with 1% aqueous aluminum phosphate and stained with aniline blue. Microscopy (Zeiss AxioX-4, Zeiss, Jena, Germany) was used to examine staining after sealing with neutral resin, and Image-Pro Plus 6.0 (ImagJ-FIJI-Trmk, Tokyo, Japan) was used to measure the positive staining area.

### 2.16. Immunofluorescence Analysis

Deparaffinization of the tissue slices was performed via proteolytic antigen retrieval (PIER) treatment via HistoReveal regent (ab103720, Abcam) at room temperature (RT) for 10 min. Then, the tissue slices were permeabilized with Triton X-100 (5%, *v*/*v*) at RT for 5 min and incubated in H_2_O_2_ (0.5%, *v*/*v*) at RT for 30 min. Each treatment was followed by washing with PBS buffer at RT 3 times for 10 min each. Next, the slices were blocked with BSA (5%, *w*/*v*) at RT for 30 min, after which immunostaining was performed. The primary antibodies used included anti-Runx2 (catalog no. ab76956; Abcam) and anti-S9.6 (catalog no. MABE1095; Millipore), and the secondary antibodies used were goat anti-rabbit IgG H&L (Alexa Fluor^®^ 488, ab150073; Abcam) and goat anti-mouse IgG H&L (Alexa Fluor^®^ 594, ab150116; Abcam). Normal rabbit IgG (catalog no. ab172730; Abcam) and normal mouse IgG (catalog no. sc-2025; Santa Cruz Biotechnology, USA) were used as negative controls. Nuclei were stained with 4,6-diamidino-2-phenylindole dihydrochloride (DAPI, Invitrogen). A microscope (Olympus, Tokyo, Japan) was used to examine the fluorescence staining.

### 2.17. Statistical Analysis

All the statistical calculations used in this study were analyzed using the SPSS 10 statistical software. Statistical significance was analyzed using one-way ANOVA or Student’s *t*-test; *p* ≤ 0.05 was regarded as significant.

## 3. Results

### 3.1. Aging-Downregulated Hmga2 Could Reduce the R-Loop Level in Aged mBMSCs

**Figure 1:**
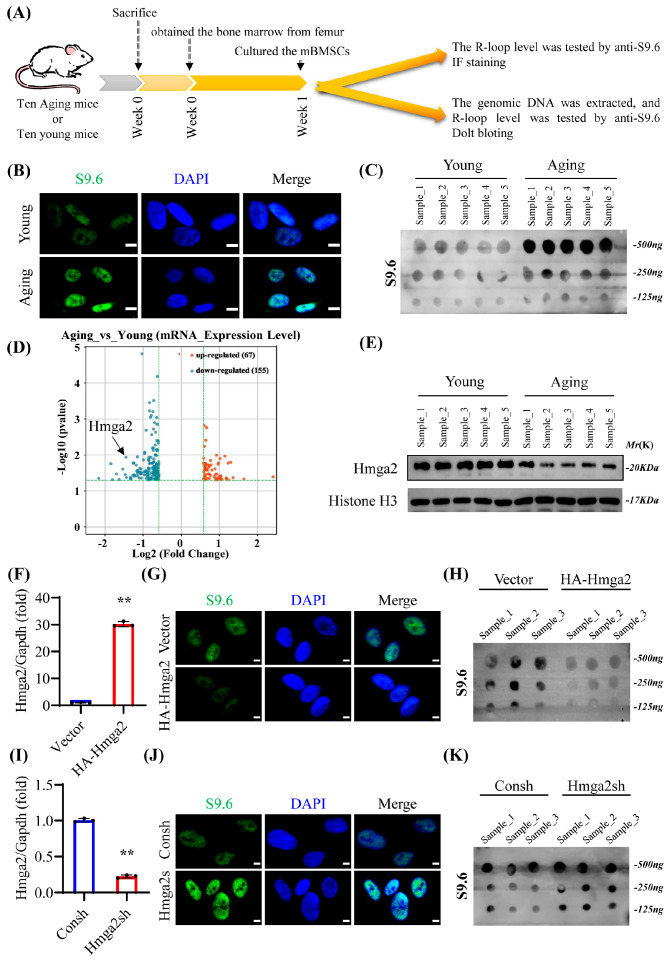
Aging-downregulated Hmga2 could reduce the R-loop level in aged mBMSCs. (**A**) Experimental design. (**B**) IF staining of R-loop (green) and nuclei (blue) in young (3 months old) and aging (20 months old) mBMSCs. Scale bar = 5 μm. (**C**) Blot results of R-loop in young and aging mBMSCs. (**D**) mRNA-Seq assay of young and aging mBMSCs. (**E**) Western blot results showing the expression of Hmga2 in young and aging mBMSCs. Histone H3 was used as the internal control (n = 5). (**F**) Efficiency of Hmga2 overexpression in aging mBMSCs. (**G**) IF staining of R-loop in Hmga2-overexpressing aging mBMSCs. Scale bar = 5 μm. (**H**) Blot results of R-loop in Hmga2-overexpressing aging mBMSCs. (**I**) Deletion efficiency of Hmga2 in aging mBMSCs. (**J**) IF staining of R-loop-containing Hmga2-depleted mBMSCs. Scale bar = 5 μm. (**K**) Blot results of R-loop in Hmga2-depleted mBMSCs. ** *p* < 0.01.

First, the immunofluorescence staining and dot blotting results reveal that the level of R-loops in aging mBMSCs was significantly greater than that in young mBMSCs (Figure 1A–C). Compared with the young mBMSCs, the aging mBMSCs presented a total of 67 upregulated genes and 155 downregulated genes, including Hmga2 (Figure 1D). Furthermore, the Western blot results reveal that the level of Hmga2 was lower in aging BMSCs than in young mBMSCs (Figure 1E). Hmga2 was subsequently overexpressed in aging mBMSCs via retroviral transduction (Figure 1F). The immunofluorescence staining and dot blotting results reveal that Hmga2 overexpression significantly repressed the level of R-loops compared with that in the control group (Figure 1G,H). Hmga2 was subsequently deleted via the transfection of a special shRNA lentivirus (Figure 1I). The immunofluorescence staining and dot blotting results reveal that Hmga2 deletion significantly increased the level of R-loops in aging mBMSCs compared with that in the control group (Figure 1J,K). These results suggest that the expression of Hmga2 was downregulated in the aging mBMSCs, which might have caused the accumulation of R-loops in these cells.

### 3.2. Deletion of Hmga2 Aggravated Senescence Marker Levels and Repressed the Osteogenic Differentiation Ability of Aging mBMSCs

**Figure 2:**
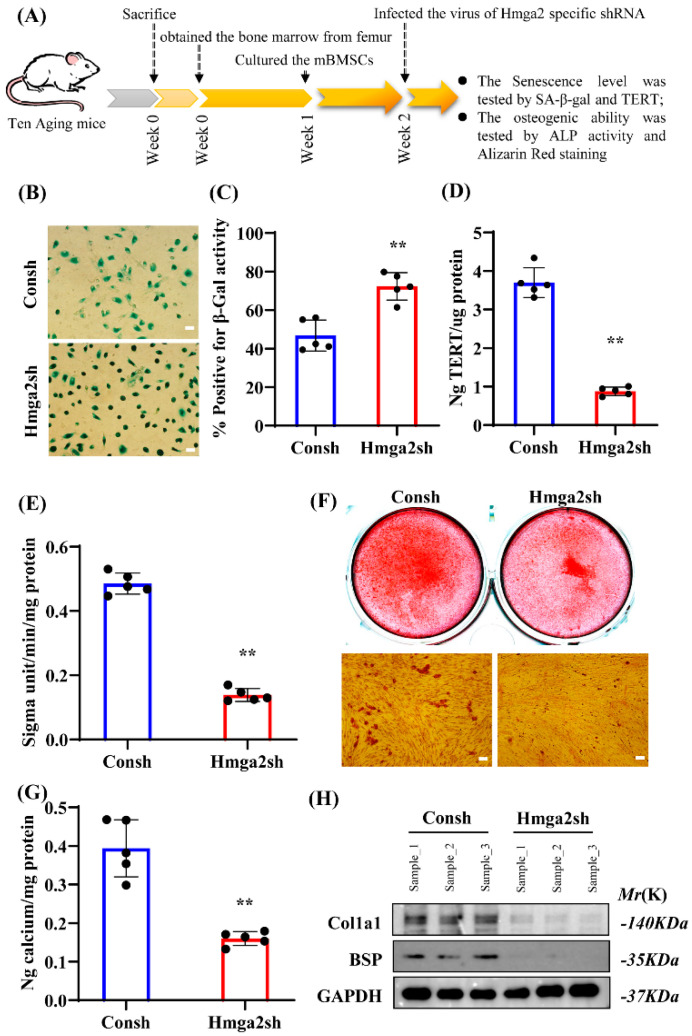
Deletion of Hmga2 increased senescence and repressed the osteogenic differentiation ability of aging mBMSCs. (**A**) Experimental design. (**B**) SA-β-gal staining of aging mBMSCs. Scale bar = 200 μm. (**C**) The percentage of SA-β-gal-positive cells. (**D**) TERT activity assay in aging mBMSCs. (**E**) ALP activity assay after osteogenic induction for 5 days. (**F**) Alizarin red staining results after osteogenic induction for 2 weeks. Scale bar = 200 μm. (**G**) Quantification of calcium levels after 2 weeks of osteogenic induction. (**H**) Western blot results showing the expression of Col1a1 and BSP in young and aging mBMSCs. Gapdh was used as the internal control (n = 3). Statistical significance was tested by Student’s *t* test. The error bars represent the SDs. ** *p* < 0.01.

To detect the effects of Hmga2 on aging mBMSCs, we focused on the senescence marker levels and osteogenic differentiation ability (Figure 2A). The SA-β-gal staining results reveal that Hmga2 deletion significantly increased the percentage of SA-β-gal-positive cells in aging mBMSCs compared with that in the control group (Figure 2B,C). TERT level analysis revealed that Hmga2 deletion significantly decreased the level of TERT in the aging mBMSCs compared with that in the control group (Figure 2D). After 5 days of osteogenic induction, the ALP activity results reveal that Hmga2 deletion significantly repressed ALP activity in the aging mBMSCs compared with that in the control group (Figure 2E). After 2 weeks of osteogenic induction, alizarin red staining and quantitative calcium analysis revealed that Hmga2 deletion significantly repressed the mineralization ability of aging mBMSCs compared with that of the control group (Figure 2F,G). Furthermore, the Western blot results reveal that the expressions of Col1a1 and BSP were significantly downregulated in the aging BMSCs after 2 weeks of osteogenic induction (Figure 2H). These findings suggest that Hmga2 is needed for BMSCs, as its deletion increased the senescence and repressed the osteogenic differentiation ability of aging mBMSCs.

### 3.3. Overexpression of Hmga2 Repressed Senescence Marker Levels and Promoted the Osteogenic Differentiation Ability of Aging mBMSCs

**Figure 3:**
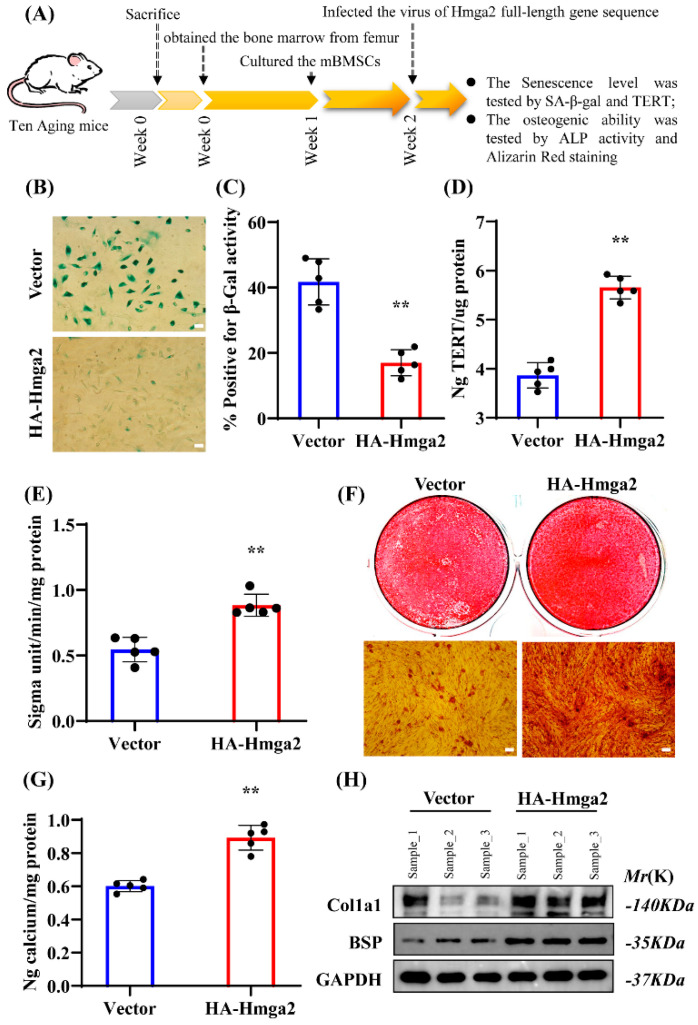
Hmga2 overexpression repressed senescence and promoted the osteogenic differentiation ability of aging mBMSCs. (**A**) Experimental design. (**B**) SA-β-gal staining of aging mBMSCs. Scale bar = 200 μm. (**C**) The percentage of SA-β-gal-positive cells. (**D**) TERT activity assay in aging mBMSCs. (**E**) ALP activity assay after osteogenic induction for 5 days. (**F**) Alizarin red staining results after osteogenic induction for 2 weeks. Scale bar = 200 μm. (**G**) Quantification of calcium levels after 2 weeks of osteogenic induction. (**H**) Western blot results showing the expression of Col1a1 and BSP in young and aging mBMSCs. Gapdh was used as the internal control (n = 3). Statistical significance was tested by Student’s *t* test. The error bars represent the SDs. ** *p* < 0.01.

The SA-β-gal staining results reveal a significant decrease in the percentage of Hmga2-overexpressing aging mBMSCs compared with that in the control group (Figure 3A–C). Additionally, the results of the TERT level analysis reveal that Hmga2 overexpression increased the level of TERT in the aging mBMSCs compared with that in the control group (Figure 3D). The ALP activity results reveal that after 5 days of osteogenic induction, Hmga2 overexpression increased ALP activity in the aging mBMSCs compared with that in the control group (Figure 3E). The alizarin red staining and quantitative calcium results reveal that after 2 weeks of osteogenic induction, Hmga2 overexpression enhanced the mineralization ability of the aging mBMSCs compared with that of the control group (Figure 3F,G). The Western blot results reveal that the expressions of Col1a1 and BSP were significantly upregulated in the aging BMSCs compared with the control BMSCs after 2 weeks of osteogenic induction (Figure 3H). These findings suggest that Hmga2 might act as a key promoter in aging BMSCs to reduce the degree of senescence and promote their osteogenic differentiation ability.

### 3.4. Hmga2 Promoted the Expression of Key Osteogenic Genes in Aging mBMSCs by Decreasing the R-Loop Level

**Figure 4:**
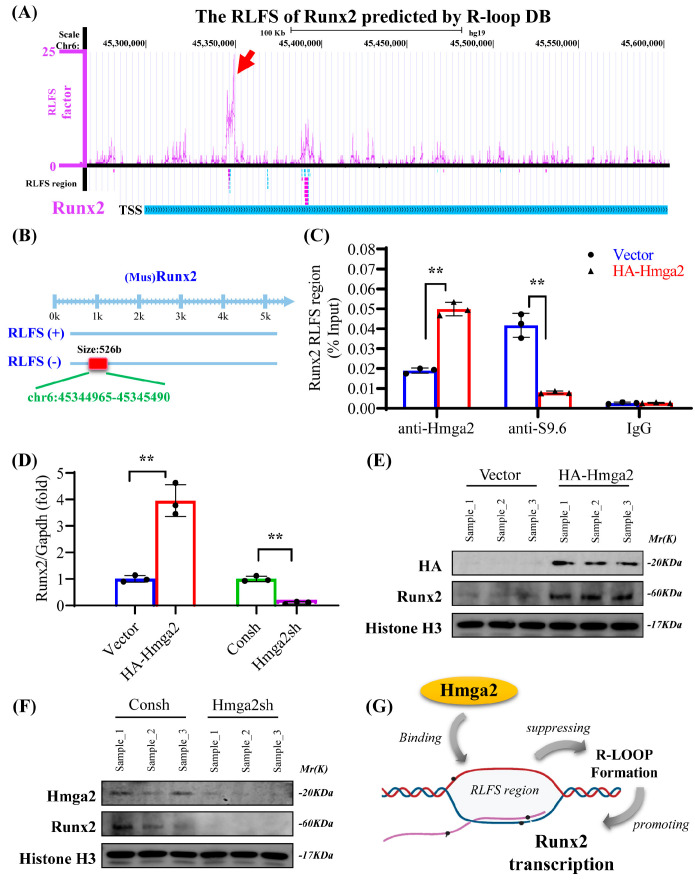
Hmga2 promoted the expression of key osteogenic genes in aging mBMSCs by decreasing the R-loop level. (**A**) R-loop-forming sequence (RLFS) of Runx2 predicted by the R-loop DB website. (**B**) RLFS of Runx2 predicted by the QmRLFS-finder website. (**C**) ChIP and DRIP assay results showing Hmga2 binding and R-loop levels in the RLFS region of Runx2. (**D**) Real-time RT-PCR results of Runx2 mRNA expression in aging mBMSCs. (**E**) Western blot results of Runx2 in Hmga2-overexpressing mBMSCs. (**F**) Western blot results of Runx2 in Hmga2-depleted aging mBMSCs. (**G**) Schematic diagram showing the regulation of the R-loop by Hmga2. Gapdh was used as the internal control (n = 3). Statistical significance was tested by Student’s *t* test. The error bars represent the SDs. ** *p* < 0.01.

To determine the underlying mechanism of Hmga2 in osteogenic differentiation, the RLFS of several key osteogenic genes, including the key transcription factors Runx2 and Osx, the early-stage differentiation marker Alpl, the collagen formation gene Col1a1, and the key bone matrix proteins Ocn and Bmp2 (Figure S1A–F), were predicted via the QmRLFS-Finder website. These findings suggest that R-loop formation sites commonly exist in the DNA stand of osteogenesis-related genes, and that changes in the R-loop level could regulate the expressions of marker genes at different times and in different spaces during osteogenic differentiation. In more detail, the key transcription factor of osteogenic differentiation, Runx2, was focused on. The RLFS of Runx2 was predicted via the R-loop DB website, and one significant R-loop formation region in the Runx2 gene was identified (indicated by red arrows; Figure 4A). We further predicted the RLFS of the Runx2 gene via the QmRLFS-finder website and revealed that the significant RLFS region was approximately 526 bp in length and located in the negative sense chain (strand-) of the Runx2 gene. The detailed sequence of the RLFS is chr6:45344965--45345490 (Figure 4B). The ChIP assay results reveal that the binding of Hmga2 to the RLFS region of Runx2 was significantly greater in the HA-Hmga2 group than in the control group (Figure 4C). The DRIP assay results reveal that the R-loop level in the RLFS region of Runx2 was significantly lower in the HA-Hmga2 group than in the control group (Figure 4C). The real-time RT-PCR results reveal that the mRNA expression level of Runx2 was significantly increased in the HA-Hmga2 group but significantly decreased in the Hmga2sh group compared with the control group (Figure 4D). Furthermore, the WB results reveal that the protein level of Runx2 was significantly increased in the HA-Hmga2 group but significantly decreased in the Hmga2sh group (Figure 4E,F). These results suggest that Hmga2 binds to the RLFS region of the Runx2 gene and decreases R-loop formation in the RLFS region of Runx2, thus increasing the expression of Runx2 and improving the osteogenic differentiation ability of aging mBMSCs (Figure 4G).

### 3.5. Hmga2 Overexpression Alleviated the Bone Loss Phenotype in Aging Mice

**Figure 5:**
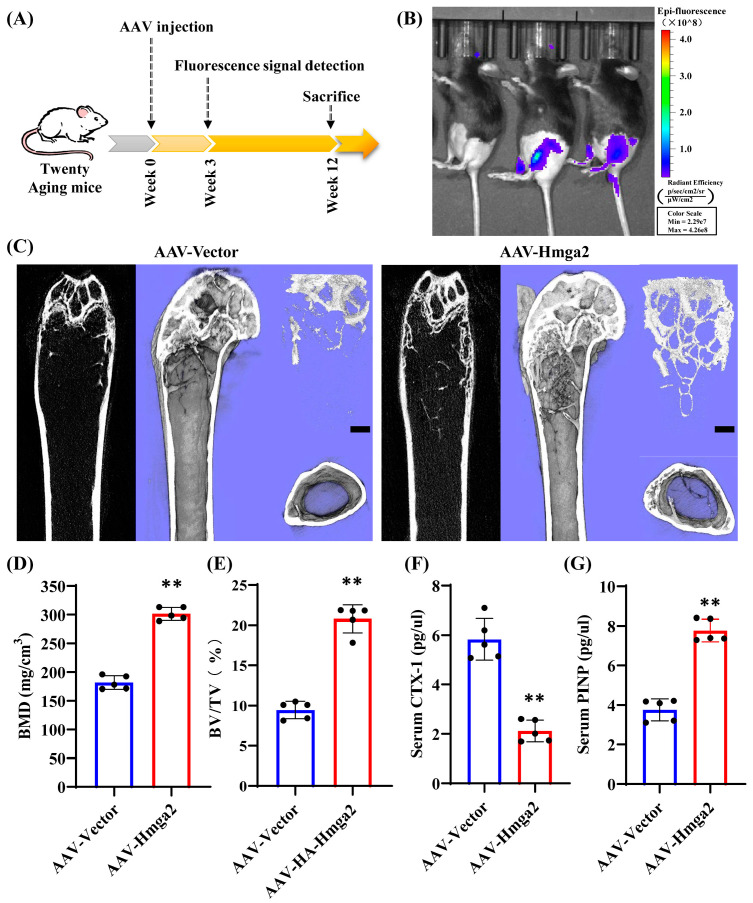
Hmga2 overexpression alleviated the bone loss phenotype in aging mice. (**A**) Experimental design. (**B**) Fluorescence signal in aging mice. (**C**) Micro-CT image and 3D reconstruction of the femur of aging mice. (**D**,**E**) Bone histomorphometry indices of BMD (**D**) and BV/TV (**E**). (**F**,**G**) The levels of CTX-1 (**F**) and PINP (**G**) in the serum were detected via ELISA. Statistical significance was tested by one-way ANOVA or Student’s *t* test. The error bars represent the SDs (n = 5). Scale bar = 250 μm. ** *p* < 0.01.

Furthermore, an AAV-expressing Hmga2 (AAV-Hmga2) was constructed and injected into the proximal joint end of the aging mice femur (Figure 5A). To verify the transfection of AAV-Hmga2, the fluorescence signal results reveal that a fluorescence signal was evident in the femurs of the injected mice (Figure 5B). In addition, the micro-CT results reveal that the bone structure of the femur cavity in the AAV-Hmga2 group was significantly greater than that in the control (AAV-Vector) group (Figure 5C). Quantitative analysis of bone histomorphometric indices revealed that the BMD and BV/TV were both significantly higher in the AAV-Hmga2 group (30.15 ± 0.91 and 20.64 ± 1.45) than that in the control group (18.16 ± 1.06 and 9.45 ± 0.98) (Figure 5D,E; Tables S1 and S2). Furthermore, the bone turnover-sensitive biomarkers in the serum were detected via ELISA, which revealed that the level of bone resorption marker CTX-1 was significantly lower in the AAV-Hmga2 group (2.12 ± 0.36) than that in the control group (5.82 ± 0.75) (Figure 5F; Table S3), and the level of bone formation marker PINP was significantly greater in the AAV-Hmga2 group (7.76 ± 0.51) than that in the control group (3.76 ± 0.50) (Figure 5G; Table S4). This indicates that the Hmga2 treatment improved bone homeostasis at the bone loss location of the aging mice.

### 3.6. Hmga2 Overexpression Upregulated Runx2 Expression and Repressed R-Loop Formation in Aging Mice

**Figure 6:**
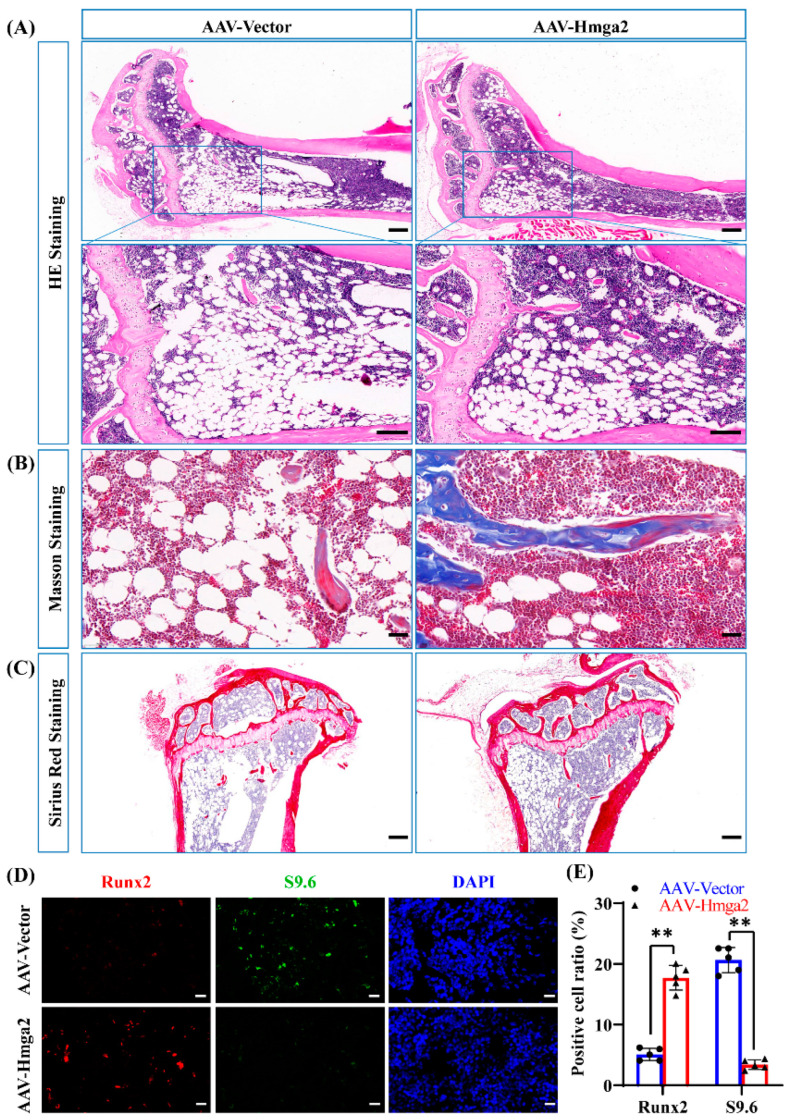
The overexpression of Hmga2 improved bone matrix remodeling in aging mice. (**A**) HE staining of bone marrow from aging mice. Scale bar = 200 μm. (**B**) Masson’s trichrome staining revealed collagen remodeling in the bone marrow of aging mice. Scale bar = 50 μm. (**C**) Sirius red staining revealed total collagen in the bone marrow of aging mice. Scale bar = 200 μm. (**D**) Immunofluorescence staining showing the level of R-loop formation (S9.6; green) and the level of Runx2 (red) in the bone marrow of aging mice. Scale bar = 50 μm. (**E**) Quantitative results of immunofluorescence staining. Statistical significance was tested by one-way ANOVA or Student’s *t* test. The error bars represent the SDs (n = 5). ** *p* < 0.01.

HE staining revealed that the number of trabecular structures and bone marrow cells in the AAV-Hmga2 group was significantly greater than that in the control group, indicating a denser and less vacuolar structure (Figure 6A). Masson staining was used to visualize collagen remodeling in the bone marrow under the growth plate (immature collagen in blue and mineralized collagen in red), and that in the AAV-Hmga2 group was significantly greater than that in the control group (Figure 6B). Sirius red staining revealed that the AAV-Hmga2 group contained more fibers, and the width of the growth plate in the AAV-Hmga2 group was wider than that in the control group (Figure 6C). Immunofluorescence staining and quantitative analysis revealed that the level of S9.6 in the bone marrow was significantly lower in the HA-Hmga2 group than in the control group (Figure 6D,E). Additionally, the expression level of Runx2 was significantly greater in the HA-Hmga2 group than in the control group (Figure 6D,E). These results indicate that Hmga2 repressed the R-loop level and upregulated the expression of Runx2 in the bone marrow of the aging mice. The Hmga2 treatment enhanced bone matrix remodeling in the aging mice.

## 4. Discussion

Aging-related bone loss leads to insufficient jawbone mass in the elderly, which affects the implementation and quality of implant surgery. Improving bone homeostasis in aging bodies still requires further solutions. As BMSCs undergo the aging process, R-loop-induced DNA replication stress unavoidably occurs during the transcription of osteogenesis-related genes. Therefore, exploring aging-related R-loop modification changes is expected to provide more evidence for improving BMSCs’ ability to generate new bones. Here, we investigated the role of Hmga2 in the osteogenic differentiation ability of aging BMSCs.

The prominent hallmark of aging cells is the accumulation of different types of stress [1]. During cell aging, R-loop formation is closely related to DNA replication stress, which is associated with DNA-binding proteins [7]. To investigate the role of Hmga2 in aging mBMSCs, we first detected several key senescence markers, including SA-β-gal and TERT. Our study revealed that Hmga2 significantly decreased the percentage of SA-β-gal-positive cells in aging mBMSCs. A previous study reported that Hmga2 expression was decreased during the repeated subculture-induced senescence of human mBMSCs [9] and human umbilical cord blood-derived MSCs (hUCB-MSCs) [11,19,20]. Hmga2 reduced the in vitro aging process of hUCB-MSCs [21]. The overexpression of Hmga2 reduced the percentage of SA-β-gal-positive cells in aging rats [12]. Additionally, the ability of miR-98-5p to inhibit the viability of preosteoblast MC3T3-E1 cells was reversed by Hmga2 overexpression [17]. These studies are consistent with our results, indicating that Hmga2 has a positive role in preventing BMSC senescence and may be a critical factor or molecular marker for altering MSC senescence.

Further, our results show that Hmga2 significantly enhanced the ALP activity and mineralization ability in aging mBMSCs and promoted the expression of the key osteogenic differentiation marker Runx2. A previous study revealed that Hmga2 is involved in the differentiation of hBMSCs [15]. Knockout of Hmga2 suppresses the differentiation of preosteoblast MC3T3-E1 cells into osteoblasts [14]. The overexpression of Hmga2 rescues the MiR-495-suppressed osteogenic differentiation of mBMSCs [16]. Additionally, the ability of miR-98-5p to inhibit the osteogenic differentiation of MC3T3-E1 cells was reversed by increasing Hmga2 expression [17]. These findings are consistent with our observed phenomena and indicate that Hmga2 can promote the osteogenic differentiation ability of aging BMSCs.

Moreover, the application of Hmga2 in aging bodies was tentatively explored using an AAV delivery system. We constructed a natural aging mouse (20 months old) model, and the ELISA results show that bone resorption marker CTX-1 was significantly decreased, while bone formation marker PINP was significantly increased after Hmga2 treatment. The histological analysis revealed that Hmga2 treatment increased the collagen remodeling in the bone marrow matrix. This study reported that Hmga2 mutation highly altered body size in both mice and humans [13]. Hmga2-/- mice exhibited a reduction in bone size [14]. Hmga2-knockout mice displayed a pigmy phenotype, whereas the overexpression of Hmga2 led to gigantism and somatic overgrowth [11]. Overexpression of Hmga2 increased new bone regeneration in a Dill-hole injury mouse femur model [16]. Although further studies are needed to investigate the effects of Hmga2 treatment on the long-term physical function of aging, these findings suggest that Hmga2 was needed to maintain the bone homeostasis of aging.

R-loop formation is closely associated with the accumulation of DNA replication stress during the aging process [1]. Excessive R-loop formation leads to replication fork collisions and transcription blockade [18]. For more details, the RLFS in the Runx2, a key transcription factor in osteogenic differentiation, was predicted [22,23,24]. R-loop formation (GC skew-triggered) is often observed between the TSS and the first exon–intron junction. This type of R-loop also produces the G-G-G-G secondary structure on the non-template DNA strand, which further stabilizes the R-loop and suppresses the gene transcription process [25,26]. Hmga2 has three distinct DNA-binding domains, each consisting of 8 or 9 amino acids, which have a high affinity for short A/T-rich sequences [11]. The RLFS region was located mainly at the transcriptional front end of the Runx2 gene. Hmga2 significantly reduced R-loop formation and upregulated the mRNA expression of Runx2. The ChIP and DRIP assays in the RLFS region of Runx2 revealed a significant increase in Hmga2 binding and a decrease in R-loop formation. These suggest that Hmga2 upregulates Runx2 expression by repressing R-loop formation in aging mBMSCs.

To confirm our hypothesis, we tested the level of Runx2 expression and R-loop formation in the bone marrow of aging mice. The immunofluorescence results reveal a decrease in R-loop level and an increase in Runx2 expression after Hmga2 treatment. The colocalization of R-loop and Runx2 did not occur simultaneously. During the osteogenic process, Runx2 is involved in the expression and maintenance of bone matrix proteins, such as Col1a1 and Ocn [23,24]. Additionally, we found that Hmga2 treatment significantly increased the total collagen content in aging mice. These findings support our observation that Runx2 expression is upregulated in bone marrow and provide a basis for further investigation of the Hmga2-regulated spatiotemporal expression patterns of osteogenesis-related genes during bone remodeling. In conclusion, our study indicates that Hmga2 might be a critical regulator of the senescence and osteogenic differentiation of aging BMSCs by affecting the formation of R-loops in the genome. Additionally, Hmga2 promotes Runx2 expression in aging BMSCs by decreasing the formation of R-loops in the RLFS region.

## 5. Conclusions

Our study investigated the effect of Hmga2 on the osteogenic differentiation of aging mBMSCs. Hmga2 was found to act as an activator of mBMSCs in aging mice and facilitates the osteogenic differentiation of aging mBMSCs through suppressing the aging-induced accumulation of DNA replication stress during R-loop formation. In this study, we explored the potential interaction mechanism between DNA replication stress and the osteogenic process in aging mBMSCs, providing a new strategy for decreasing bone loss in aging individuals.

## Data Availability

The datasets used and/or analyzed during the current study are available from the corresponding author upon reasonable request.

## Supplementary Materials

The following supporting information can be downloaded at: https://www.mdpi.com/article/10.3390/medicina61050820/s1, Figure S1: The R-loop-forming sequences (RLFSs) of key osteogenic genes.; Table S1: The numerical data of the level of BMD (g/cm^3^); Table S2: The numerical data of the level of BV/TV (%); Table S3: The numerical data of the level of Serum CTX-1 (pg/µL); Table S4: The numerical data of the level of Serum PINP (pg/µL).
