# Supplementary figures and images for: Investigating the Associations Between Hmga2 Overexpression, R-Loop Reduction, and Bone Loss in Aging Mice
Source: Medicina (Kaunas). 2025 Apr 29;61(5):820. doi: 10.3390/medicina61050820 (PMC12113323; doi:10.3390/medicina61050820)

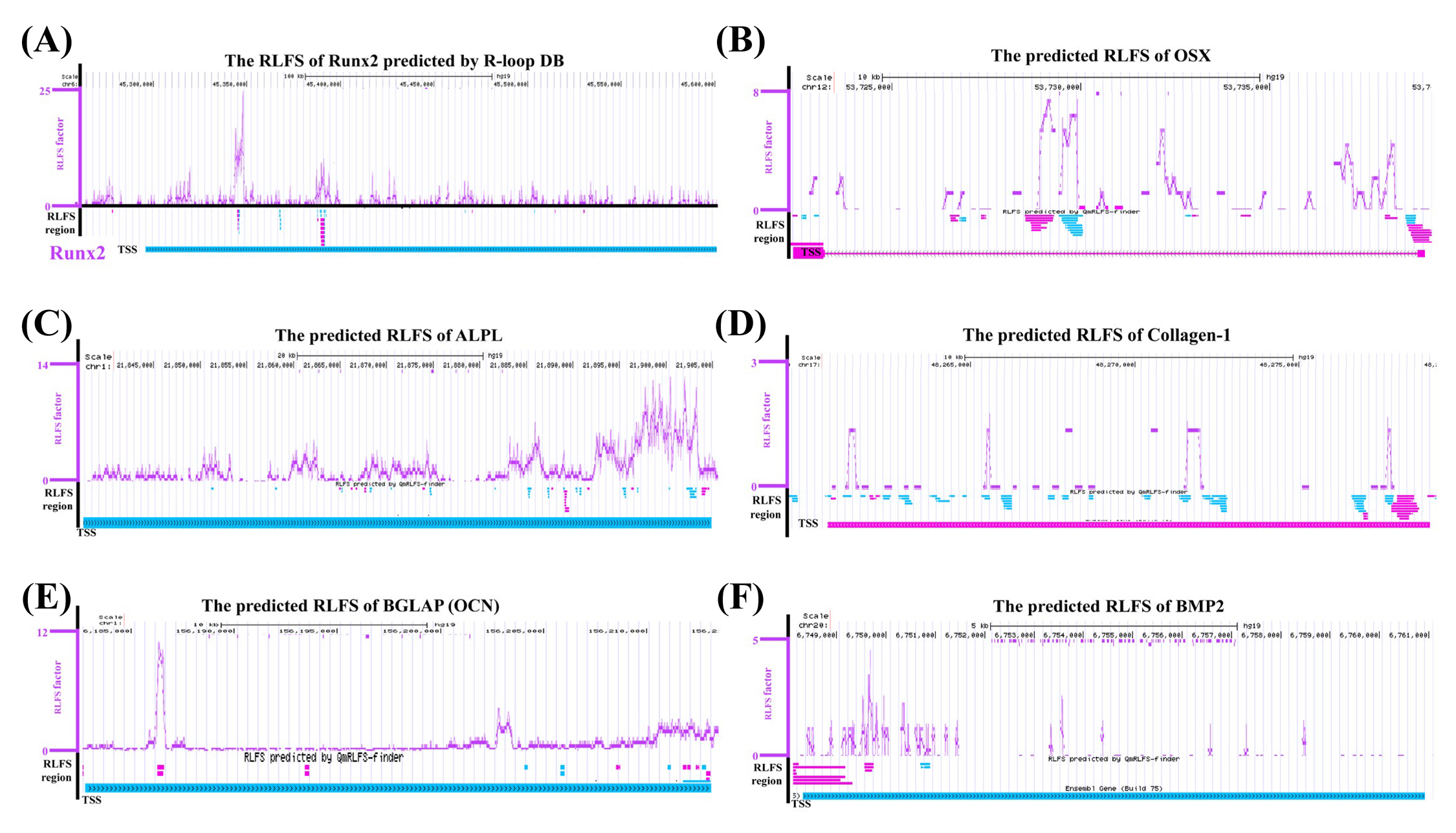

Supplement: Supplementary file 1 [file medicina-61-00820-s001.zip › Supplementary Figure_1.jpg]
